# End-of-life care planning for ethnically diverse kidney patients in the COVID-19 era

**DOI:** 10.1186/s12882-026-04866-5

**Published:** 2026-03-11

**Authors:** Zoebia Islam, Fawn Harrad-Hyde, Sherna F. Adenwalla, Matthew P. M. Graham-Brown, Christina Faull

**Affiliations:** 1 LOROS Centre for Excellence, Leicester, UK; 2 Department of Population Health Sciences, University of Leicester, Leicester, UK; 3 National Institute for Health Research (NIHR) Leicester Biomedical Research Centre, University Hospitals of Leicester NHS Trust and University of Leicester, Leicester, UK; 4 Department of Cardiovascular Sciences, University of Leicester, Leicester, UK

**Keywords:** Covid-19, Ethnically diverse, End-of-life care, End-stage kidney disease

## Abstract

**Background:**

End-stage kidney disease (ESKD) is most prevalent amongst people from minority ethnic groups and people receiving haemodialysis were considered to be ‘clinically extremely vulnerable’ during the Covid- 19 pandemic. Covid-19 impacted the health of those from ethnically diverse backgrounds disproportionally. The Covid-19 pandemic brought death and dying to the forefront of public discourse, yet few studies have explored the impact of the Covid-19 pandemic on individual’s understanding of and willingness to engage in end-of-life care planning conversations. In addition, few studies have explored end-of-life care planning in the context of ESKD, and even fewer studies have considered the complex dynamics of ethnicity and end-of-life care planning in ESKD. In this study we explored the experiences of an ethnically diverse group of patients, care givers, and healthcare professionals, who had engaged in end-of-life care planning discussions during the Covid-19 pandemic.

**Methods:**

In this qualitative study, 14 ethnically diverse patients (Indian, Pakistani and African Caribbean), including 11 male with ESKD on haemodialysis, 15 bereaved family caregivers including 11 male and four were female from Black British, Indian and Latino backgrounds and 6 healthcare professionals took part in a semi-structured interview, either face-to-face or remotely. Data were analysed thematically using Braun and Clarke framework.

**Results:**

This paper presents seven key themes. Four themes describe the experiences of patients and bereaved family caregivers and suggest that family caregivers were a key source of information and support for patients, yet they had differing levels of understanding about the nature and prognosis of ESKD. Patients and bereaved family caregivers described limited opportunities to engage in discussions about future care and a limited awareness of both the ReSPECT form and discussion process and instead, often made sense of ESKD in light of their faith. A further two themes describe the experiences of healthcare professionals suggest that healthcare professionals see end-of-life care planning as necessary but difficult and stress the need for further training. A final seventh theme describes a shared perception, amongst patients, bereaved family caregivers and healthcare professionals regarding the lack of impact of Covid-19 on end-of-life care planning in the context of supporting ethnically diverse people living with ESKD.

**Conclusions:**

Further work is needed to explore how best to improve understanding and engagement with end-of-life care planning discussions in dialysis patients from diverse ethnic backgrounds.

**Supplementary Information:**

The online version contains supplementary material available at 10.1186/s12882-026-04866-5.

## Background

End stage kidney disease (ESKD) is more common in patients from minority ethnic backgrounds, than the white British population. This is, in part, due to the increased prevalence of co-morbidities such as type 2 diabetes and hypertension in these patient groups [1]. Given the complex medical profile of patients with ESKD, during the Covid-19 pandemic these patients were classed as ‘clinically extremely vulnerable’, reflecting their increased vulnerability to deterioration and dying if they became infected (Department for Health and Social Care, [2]). Unlike other clinically vulnerable groups, many patients with ESKD required haemodialysis in healthcare settings, which limited their ability to shield and increased their chances of contracting Covid-19 [3].

At face value, it might be easy to assume that increased public awareness of death and dying during the pandemic may have promoted some people to engage in end-of-life care planning (EOLCP). There are many ways that people may engage in EOLCP. For example, individuals may engage in advance care planning (ACP), which provides an opportunity for people to express their wishes about many things, including care and treatment in the future, should they subsequently lose capacity. As part of ACP, EOLCP describes the process of shared decision-making discussions between patients, their family caregivers and healthcare professionals (HCPs). The aim of ACP is to support adults to understand and share their personal values and preferences regarding future medical care for serious deteriorations in their health [4]. Wishes may be documented in a variety of ways. Some centre around documentation of patient legally binding preferences, such as Advance Decision to Refuse Treatment orders. Others outline shared or best interest recommendations regarding emergency treatment, for example via tools such as the Recommended Summary Plans for Emergency Care and Treatment (ReSPECT) form. In many regions of the UK, the ReSPECT form is central to end-of-life care planning. Introduced by the Resuscitation Council UK in 2016, it is designed to capture shared decisions between a patient (or their representative if they lack capacity) and a senior clinician. The process involves establishing an understanding of the person’s current health, discussing how it may change, identifying preferences and goals of care, agreeing on whether the emphasis should be on life-sustaining treatment or comfort, and making specific recommendations about interventions including cardiopulmonary resuscitation (CPR) [5]). The document is standardised, portable, and stored in both paper and electronic health records, ensuring accessibility across care settings. Although not legally binding, it carries significant ethical and professional authority.

Studies on EOLCP in patients with ESKD have rarely focussed specifically on patients from minority ethnic backgrounds and patients with limited English are often excluded from research studies [6, 7]. One notable exception is the ‘Thinking Ahead’ study, [8], which explored the barriers and enablers to EOLCP with ethnically diverse patients (including those with a terminal illness or disease such as kidney disease), their family caregivers and the HCPs who supported them. The authors suggested that perceptions of ‘good’ end-of-life care were subjective and influenced by the intersectionality of diverse factors, including beliefs and culture. Furthermore, the authors found that HCPs may need additional skills and knowledge to support patients from different ethnic backgrounds to engage in EOLCP.

It is widely recognised that the changing landscape of health risks associated with Covid-19 have impacted disproportionally on those from ethnically diverse backgrounds, particularly those with haemodialysis-dependent ESKD [3]. Yet, to our knowledge, there has been little exploration of the impact of Covid-19 on understanding and readiness or willingness to engage in EOLCP conversations. This study (The CRISIS study) addressed this gap and aimed to understand whether and to what extent Covid-19 had an impact on EOLCP for ethnically diverse patients with ESKD. Here, the experiences of ethnically diverse patients with haemodialysis-dependent ESKD, bereaved family caregivers (BFCGs) and healthcare professionals’ (HCPs) were explored. In this paper the term ‘ethnically diverse’ (ED) is used to describe diversity in language, culture, religion and ethnic background including White minorities [9].

## Methods

### Study design

This qualitative exploratory interview study was based in Leicester and Leicestershire (UK), in which 59.1% of the population describe their ethnicity as ‘not white’ (Office for National Statistics, [10]). The study ran between May 2023 and March 2024. Ethical approval was obtained from East of England - Essex Research Ethics Committee on 17/01/2023, (Reference: 22/EE/0255).

In this study, three discrete groups were interviewed. These were: ED patients with ESKD on haemodialysis; BFCGs of ED patients with ESKD on haemodialysis; and HCPs working with ED patients with ESKD on haemodialysis. Recruitment and data collection processes for each group are described below. The inclusion / exclusion criteria for each group are listed in Table 1. Informed consent was obtained prior to each interview, and demographic data was collected using Case Report Forms. Interviews were audio recorded and transcribed verbatim. Participants received a £20 voucher for their time.

**Table 1:** Inclusion criteria

Participant type	Inclusion criteria
All participants	• 18 years or older • Able to comply with the study requirements • Able to give informed consent* *A person will be deemed able to give informed consent providing they can: • Understand the purpose and nature of the research • Weigh up the benefits and risks of taking part • Retain the information long enough to make decisions • Communicate their decision (acknowledging that it is the responsibility of the research team and, specifically, the person obtaining consent, to ensure that all participants are fully supported to communicate their decision).
Patients	• Ethnically diverse (including white minorities) • Living with end stage kidney disease (sometimes referred to as stage 5 chronic kidney disease) • Currently under the care of renal services at the research site
Bereaved Family Caregivers	• Bereaved (in the previous 3–36 months) of a person from an ethnically diverse background who died as a result of end stage kidney disease during the COVID-19 pandemic. At the time of death, the deceased patient was dialysis dependent. The cause of death may have been directly or indirectly related to dialysis treatment, including withdrawal or associated complications, but this was not a requirement for inclusion.
Healthcare Professionals	• Employed by research sites • Caring for ethnically diverse patients with end stage kidney disease

### Ethnically diverse patients with end stage kidney disease

Interviews were carried out with ED patients with ESKD on haemodialysis who may have had EOLCP conversations or been hospitalised during the Covid-19 pandemic. Patients were recruited from one of the three dialysis units in Leicester and Leicestershire. Potential participants were identified by a clinical member of the research team (SA). If the patient was able to discuss the study in English, SA explained the study and provided a Participant Information Sheet. If interested, the potential participant was asked to complete a Consent to Contact form. If the participant was unable to speak English, the trilingual Chief Investigator (ZI) was able to discuss the study over the telephone in the patient’s preferred language. Professional interpreters were also available for patients who spoke other languages, but their services were not required. If interested, a date and time for interview was arranged. In total, 26 patients were approached. Seven patients chose not to participate, one patient died before they took part and a further four patients were contacted multiple times with no success.

In total, 14 patients were recruited. All interviews were conducted in the preferred language of the participant by three experienced researchers two of whom were from British Asian backgrounds and the other from a White British background: Participants were unknown to the researchers prior to the interviews. Interviews were conducted for up to 60 min, which included time taken for consent. Rapport was established through culturally sensitive communication, the use of participants’ preferred language, clear explanations of the study aims, and allowing time at the start of interviews for informal conversation and questions. Two patient interviews were conducted in Hindi and two in Punjabi by ZI a experienced postdoctoral researcher with expertise in sensitive interviews; all others were conducted in English by two experienced members of the research team. Demographic details of these participants are detailed in Table 2. Two interviews were undertaken in the patients’ homes, one over the telephone and all others took place face-to-face in the renal unit whilst the patient was undergoing haemodialysis, as per patient preference. In the renal unit, measures were taken to uphold patient privacy, including the use of privacy curtains around hospital beds during the interviews. During the interview, participants were asked questions about: their experience of living with ESKD and the impact of Covid-19; their understanding of illness and prognosis; their experiences of conversations with HCPs; and the extent to which the Covid-19 pandemic influenced such discussions.

**Table 2:** Demographic information of patient participants

Pseudonym	Gender	Age	Ethnicity	Generation of migrant	Country of birth	Religion
P1	Female	75	Indian	1st	Kenya	Hindu
P2	Male	64	Indian	1st	India	Hindu
P3	Male	68	Indian	1st	Kenya	Hindu
P4	Male	56	Indian	1st	Tanzania	Hindu
P5	Male	62	Indian	1st	Tanzania	Hindu
P6	Female	62	Pakistani	1st	Pakistan	Muslim
P7	Male	45	Indian	1st	India	Hindu
P8	Female	58	Indian	1st	India	Muslim
P9	Male	26	Indian	2nd	India	Hindu
P10	Male	63	Indian	1st	India	Hindu
P11	Male	73	Indian	2nd	India	Muslim
P12	Male	63	Indian	2nd	Kenya	Hindu
P13	Male	65	Indian	1st	Kenya	Muslim
P14	Male	68	African Caribbean	2nd	Monserrat	Christian

### Bereaved family caregivers

Interviews were carried out with BFCGs of ED patients who had ESKD. BFCG participants had experienced the loss of a family member in the previous 3–36 months and were not related to any patients recruited to the study. They were recruited from across the UK via social media (*n* = 14), recommended by other participants (*n* = 2) and through researcher contacts (*n* = 1). All interviews were conducted online via Microsoft Teams. Time taken for interviews with BFCGs was again up to 60 min and rapport was established in the same way as with patient interviews. During the interviews, participants were asked to retrospectively reflect on their experience of supporting a patient with ESKD. Participants were asked questions about: their understanding of the person’s illness; their experience of being involved in conversations about future care, decision-making and treatment preferences; and their views on the extent to which the Covid-19 pandemic had influenced their discussions.

Seventeen BFCGs expressed an interest from whom 15 BFCGs were recruited. The age of the BFCGs ranged between 24 and 52 years. Eleven were male and four were female. The majority described their ethnicity as Black British (*n* = 11), two described their ethnicity as Bangladeshi, one as Indian and one as Latino, and they described their faith as Christian (*n* = 12), and Muslim (*n* = 3). Six of the BFCGs were adult children, with others being siblings (*n* = 3), grandchildren (*n* = 2), a girlfriend (*n* = 1), an uncle (*n* = 1), an aunt (*n* = 1) and a cousin (*n* = 1). Demographics of the deceased person that they cared for are detailed in Table 3. Many of the deceased people died in hospital (*n* = 11) with others (*n* = 4) dying at home.

**Table 3:** Demographic information of deceased family members of bereaved family caregiver participants

Pseudonym	Deceased patient demographic details				
	Gender	Age	Ethnicity	Generation of migrant	Place of death
BFCG1	Male	26	Black British	2nd	Hospital
BFCG2	Male	17	Black British	3rd	Home
BFCG3	Male	45	Indian	2nd	Hospital
BFCG4	Female	65	Black British	1st	Hospital
BFCG5	Male	60	Black British	1st	Hospital
BFCG6	Female	40	Black British	1st	Home
BFCG7	Female	27	Black British	Not known	Hospital
BFCG8	Male	56	Black British	1st	Hospital
BFCG9	Male	23	Black British	3rd	Leicestershire
BFCG10	Female	58	Latino	1st	Home
BFCG11	Male	79	Bangladeshi	1st	Hospital
BFCG12	Female	60	Black British	4th	Hospital
BFCG13	Male	72	Black British	3rd	Home
BFCG14	Male	54	Bangladeshi	1st	Hospital
BFCG15	Male	31	Black British	3rd	Hospital

### Healthcare professionals

Interviews were carried out with HCPs to explore their experiences of EOLCP conversations (including the use of the ReSPECT form or similar tool) with ED patients with ESKD and/or their families during and after the onset of the Covid-19 pandemic in order to determine whether and to what extent the Covid-19 pandemic altered EOLCP. Forty-five HCPs – including doctors, registered nurses, physiotherapists, occupational therapists, and healthcare assistants who worked with ED patients with ESKD within the University Hospital Leicester Renal units - were identified and contacted by clinical team members via email in the first instance.

Six HCPs were recruited and their demographic information is available in Table 4. Interviews were conducted face-to-face (*n* = 2), via Microsoft Teams (*n* = 2) and via the telephone (*n* = 2). Participants were asked about their experiences of: supporting ED patients with ESKD; EOLCP conversations with ED patients with ESKD and their families; the impact of Covid-19 on such conversations; and views on further support to improve EOLCP conversations with patients and families.

**Table 4:** Demographic information of healthcare professional participants

Pseudonym	Gender	Ethnicity	Religion	Job role
WP3-HCP1	Male	White	No religion	Consultant
WP3-HCP2	Male	Non-White British	No religion	Registrar
WP3-HCP3	Female	Non-White British	No religion	Consultant
WP3-HCP4	Female	White British	Christian	Nurse
WP3-HCP5	Female	Non-White British	Hindu	Nurse
WP3-HCP6	Female	White British	Christian	Nurse

NB: Ethnicity is described as White and Non-White British to protect anonymity

### Data analysis

Data were analysed thematically [11, 12]. Each dataset was initially analysed separately. Two researchers (ZI and FHH) familiarised themselves with the data by reading and re-reading the transcripts. Whilst both researchers had experience of conducting research focused on palliative and end-of-life care, and ZI had experience of conducting research focussed specifically on ethnically diverse populations, neither had previous experience conducting research with or working with people living with ESKD. Each researcher’s previous experience provided sensitising concepts which could be explored during analysis. However these were used as ‘directions along which to look’ ([13], p7) as opposed to a framework which guided analysis. Then, each researcher undertook a process of ‘open coding’ independently, using NVivo version 20, allocating segments of each transcript to one or more codes. Next, themes were identified through an iterative process of looking across initial codes and discussing, across the wider research team, how codes may relate to one another both within and across interviews. Data saturation was reached when continued coding produced no additional concepts or interpretive insights, and the research team agreed that further analysis would not enhance the thematic development. Once initial themes had been identified, the three datasets were combined. Through a process of constant comparison, the authors were able to identify similarities and differences between the datasets and themes were refined and finalised.

### Public and patient involvement

The study was developed with patient and public involvement (PPI) including PPI Research Consultees linked to LOROS hospice. Here, input was sought from a group consisting of up to 10 individuals which comprised of ED members of the public, carers and bereaved relatives (referred to as the PCBR group). Throughout the course of the study the PCBR group met three times and provided input into the development and progress of the study. For example, they discussed and informed the development of the study’s design and proposed methodology to ensure recruitment practices were appropriate throughout. This was essential due to the potentially sensitive nature of the interviews.

Topic guides were initially developed by the research team based on a review of existing literature. However, the PCBR group also provided advice about the proposed materials that were developed to support recruitment and data collection (such as topic guides and participant information sheets) which enabled the research team to ensure that such materials were developed and implemented in a way that was feasible and acceptable to potential participants.

Following this, pilot interviews were conducted with three patients with ESKD who were part of an existing PPI group for patients on haemodialysis to further check the appropriateness of content and language of the topic guides. At later stages of the project, the PCBR group were presented with selected interview excerpts and emerging themes from patients, BFCGs, and HCPs. PCBR members reviewed these materials and discussed their interpretations, focusing on what participants considered important when discussing death, dying, and end-of-life care planning, as well as factors that facilitated or hindered these conversations. Their reflections and feedback on the initial findings contributed to refining and strengthening the research team’s interpretations.

## Results

The results below describe the key themes, which represent ED patients with ESKD, BFCGs and HCPs experiences of engaging in EOLCP during and after the Covid-19 pandemic. Themes are reproduced and organised in Table 5. Here, the first four themes relate to the experiences of patients and BFCGs and are presented together. The following two themes related to the experiences of HCPs and a final seventh cross-cutting theme represents all three datasets.

**Table 5:** Participant experiences of engaging in EOLCP during and after the COVID-19 pandemic

Patient and Bereaved family caregiver Experiences	Healthcare professional experiences
1. Misunderstandings around prognosis in end stage kidney disease 2. A lack of discussion about deterioration and future care 3. A lack of awareness of the ReSPECT form and discussion process 4. Making sense of illness, deterioration and end-of-life in light of faith	5. End of life care planning as necessary but difficult 6. A desire for further training

Shared experiences across all participant groups	
7. Lack of impact of covid-19 on end- of-life care planning conversations	

### Patients with end stage kidney disease and bereaved family caregiver experiences

#### Misunderstandings around prognosis in end stage kidney disease

Many patients and BFCGs were not aware of the severity of ESKD. Only four patient participants and two BFCGs said that they understood there was “no cure” for ESKD, and that unless deemed eligible for a transplant they would require lifelong dialysis. Three patients said they did not understand ESKD. Eight participants (three patients and five family members) seemed to have unrealistic expectations of the impact of dialysis – suggesting that dialysis might help the person to ‘get better’.

*[Interviewer: What’s your understanding of end stage kidney disease? ] Me don’t get much*,* so I don’t understand much about it.*

*[Interviewer: You don’t? ] No. Me don’t understand much about it. (P14)*

*The only future he was expecting was when he would be 100% fit. So that was the only future I had for him.*

*[Interviewer: OK*,* you had an understanding that he may get better? ]*

*Participant: Yeah. (BFCG15)*

Family caregivers were key sources of practical and emotional support for patients. They were also informal sources of information for patients, often providing information about the person’s illness and treatment. Many attended clinical appointments with HCPs, particularly where there were language barriers:

*Only my wife and my children*,* they help me if I need them*,* that’s all. Otherwise*,* I don’t go other people for extra help*,* no… If I’m tired*,* they help me to take me in the bath…Sometimes they drive me if I can’t drive. (P5)*

#### A lack of discussion about deterioration and future care

The majority of patient and BFCG participants who reported discussions with HCPs suggested that their discussion centred around the impact of lifestyle factors, such as diet and exercise (six patients) and potential treatment options, for example suitability for a transplant or dialysis (two family members). However, the remaining participants reported little opportunity to discuss future prognosis with healthcare professionals. Some family members expressed a desire to have better or ‘more proactive’ conversations with healthcare professionals

*The healthcare professionals… they’re not really proactive*,* they’re like reactive… I think they should have made more effort about it…. They could have a one-to-one discussion to say this*,* dad’s declining. We did discuss but not kind of about end-of-life. (BFCG11)*

However one patient participant did not have a desire for further discussions with healthcare professionals.

*I’ve not talked to healthcare. I don’t want to talk to healthcare unless when I need to talk to them. Because up to now everything is OK. (P13)*

#### A lack of awareness of the ReSPECT form and discussion process

The majority of patients and BFCGs had limited health and death literacy. For instance, the majority of patient participants were unaware of the ReSPECT form and therefore did not understand what this entailed and how it formed part of EOLCP conversations. Two patient participants reported they had a ReSPECT form, however details of this were only recorded in one of the two patients’ medical notes. Three patients who did have a ReSPECT form in their medical notes appeared to be unaware that one was in place.

*No*,* no*,* there was no discussion like that*,* nothing about the future. No*,* there was never any discussion like that. (P10)*

Of the 15 BFCG participants interviewed, eight had no knowledge of ReSPECT. Four had ‘heard’ of ReSPECT but believed this had been completed by another family member. One believed they had completed a ReSPECT form on behalf of the family member. One believed they had completed the ReSPECT form but could not remember what the discussion entailed. One could not remember whether a form had been completed. Some participants interpreted the lack of conversation as a sign that healthcare professionals believed the person’s condition was stable and therefore they were unlikely to deteriorate.

*I would assume that the doctors were fairly confident that his condition was stable and they had no fear of anything that is happening (BFCG14).*

#### Making sense of illness, deterioration and end-of-life in light of faith

The majority of participants - nine patients and eight BFCGs - appeared to make sense of their illness in light of their faith. For example, two suggested that their life was being sustained by *“medications and prayers to my God”* (P2). However, participants tended not to discuss their beliefs with HCPs because of their own perceived understanding of HCPs beliefs:

*She was also somebody who used to believe*,* her faith was important to the journey.*

*[Interviewer: Do you remember whether the healthcare professionals considered your aunt’s faith when thinking ahead about her illness? ]*

*I don’t think so because some of the healthcare providers*,* they don’t believe in the existence of God and all that. So*,* I don’t think my aunt’s faith mattered to them. (BFCG6)*

When asked whether HCPs considered their faith, participants provided mixed responses. Some felt that HCPs *did* consider faith, for example, noting the person was given a bible near to their death or that staff ‘prayed for’ or ‘with’ the person. However, two participants reported they were not asked about their faith and a further two reported they were asked but they did not feel that their faith had been taken into consideration during discussions. Others suggested that HCPs considered faith ‘to an extent’, recognising that HCPs may be obliged to offer and discuss treatments that contradicted with the person’s faith:

*He is professional doctor*,* so he’s more or less in the medical side… he does consider individual people with their faith… but he will put in front of me what dangers are there…So it’s entirely up to you whether you want to stick to your faith or whether you want [medical advice]. (P3)*

In addition, one participant suggested there ‘wasn’t anything to consider’ because their faith did not conflict with medical advice.

*When it came to our meetings there was nothing – there was no kind of conflict*,* if you get what I mean? So*,* there wasn’t even anything to consider*,* if I’m being honest. (BFCG14)*

### Health care professional experiences

#### End-of-life care planning as necessary but difficult

All six healthcare professionals acknowledged the importance of having EOLCP conversations and shared examples of how they broached EOLCP conversations with patients and their families, for example, by saying *“you’re relative has survived much longer than we expected*,* but there does come an endpoint for us all”* (HCP1), or by informing patients and families that dialysis is not a cure for ESKD. Another HCP said they built up to talking about EOLCP, introducing the concept of resuscitation and then moving to talking about ‘how far’ to take treatment, outlining the potential benefits and burdens of different options:

*We go on to have a conversation about how far we’re going to take their care*,* because we want to do everything for them but [all treatments] are a double-edged sword. Everything comes with consequences. And it’s about understanding what’s acceptable to them as a person. (HCP2)*

Despite acknowledging the importance of EOLCP, all six HCPs described EOLCP conversations as ‘difficult’. Sources of difficultly included: concerns that there was not sufficient time to discuss EOLCP; disagreement amongst family members and instances in which families may be “in denial” (HCP5 and HCP6) and unwilling to discuss the notion that the person would not get better.

*We don’t do it as often as we should and mainly because of how difficult the conversation is itself*,* plus lack of time…to do it properly*,* you need an appropriate environment*,* preferably with people who they want to be there during the discussion. (HCP3)*

*It’s easier to have those conversations with the families around. There’s some exceptions*,* particularly when there’s conflict between… when the patient wants to stop but the family don’t want them to stop. (HCP1)*

*They are very much thinking that this is all going to pass and trying to open up that conversation has been really challenging… it doesn’t matter how you put it across. (HCP6)*

It was clear that HCPs were reluctant to broach EOLCP and ReSPECT conversations unless the patient or their family broached this. They were also hesitant to use the words ‘death’ or ‘dying’ with patients and families for fear of upsetting them. This appeared to be reflected in participants interview responses, in instances where participants refrained from using the word ‘die’. For example, one participant said: *“Families do understand that this form means there’s no*,* yeah*,* most of the time they understand”* (HCP5). In addition, another said *“because they do understand that*,* you know*,* there would be a limited time that they will have after they stop dialysis”* (HCP3).

#### A desire for further training

All HCPs acknowledged the time and skills EOLCP required. Furthermore, all HCPs acknowledged the potential negative consequences of occasions when EOLCP might not ‘go well’. As a result, all HCPs welcomed further training and support.

*When it goes well it can be very rewarding for myself as a clinician. But equally*,* if it doesn’t go well*,* it can be really devastating for the family but also for yourself… The more guidance we can have… I think would be helpful and I think that we need to be better at it. (HCP1)*

The majority of HCPs (five) had received training around communication and managing sensitive conversations, delivered by the local hospice. All who had attended such training suggested they had found it beneficial. However, HCP participants also discussed the need for further training on a range of topics, including communication skills specifically related to EOLCP within the context of ESKD. For example, participants suggested that they would appreciate training on topics such as how to deal with conflict management during EOLCP, how to have EOLCP conversations with people who may be in ‘denial’ or who speak a different language, and training tailored specifically to EOLCP within the context of ESKD.

*I think there should be more [training]… a few more tools in the bag… to support HCPs with that planning because where do you get those skills from if you’ve not had formal training? It’s just life experience… and being able to read people’s needs and read between the lines. (HCP6)*

### Shared experiences across all participant groups

#### Lack of impact of Covid-19 on end-of-life care planning conversations

Participants across all three groups suggested that Covid-19 had not had an impact on the experiences of EOLCP. Only a small minority of patient participants (five) reported they had discussions about the potential impact Covid-19 could have on the health of people living with ESKD. Conversations about Covid-19 tended to centre around the importance of preventative measures. For example, one participant stated *“They just tell me to wear a mask*,* you know*,* keep the distance*,* use the sanitiser*,* that’s all”* (P5). Three patient participants reported being encouraged by HCPs to have vaccinations, yet one described a desire for a greater level of information about discussion about the potential benefits and burdens of vaccinations for people living with ESKD.

We were told to go and make an appointment for the injection, the vaccination, and then we did, that’s all. They just say “Have it. Have it” but what for? Why for? You know, [we were] not fully informed. What side effect do we have, as a kidney patient? I don’t know. (P6)

The remainder of the patient participants reported that they had not discussed the potential impact that Covid-19 could have had on them and their health, nor the possibility that catching Covid-19 could lead them to experience a potentially serious deterioration in their health.

*I know that Covid is one of those things that if you were to get it*,* you’d get worse. But no-one’s spoken to me about that. (P10)*

This was also replicated in interviews with BFCGs. A minority (four) suggested that they had discussed the impact that Covid-19 might have on the health of the person living with ESKD, specifically the increased likelihood that the person would deteriorate and die. A further three stated that they had discussed precautionary measures (such as vaccinations and the importance of isolating) with healthcare professionals, yet the majority of participants did not report having conversations about the impact of Covid-19 with HCPs.

*They were just telling me that if my brother gets infected with Covid-19 that it would be easy for him*,* like the chance of him being alive would be*,* let’s say*,* 10%. (BFCG15)*

Whilst HCP participants felt that Covid-19 had influenced their work, for example through the introduction of virtual consultations and additional infection control measures, many said it had not altered their EOLCP discussions with patients and families. Furthermore, HCPs recognised that the additional effort put into EOLCP during early phases of Covid-19 had not been sustained:

*We are not doing [EOLCP] as frequently as we were during Covid-19 … people want to keep that as far back in their minds as possible… I think generally there should be more information about it*,* not only in hospitals. (HCP3)*

When probed further about the lack of impact that Covid-19 had on the experiences of EOLCP, several participants highlighted the presence of competing priorities. Furthermore, one participant suggested that they could not see the relevance of discussing Covid-19 in the context of end-of-life care and ESKD:

*I’d like to think so but I’m not convinced… during the first few waves we were much more systematic about getting ReSPECT forms in place. But I think that’s perhaps fallen down the list or priorities. (HCP1)*

*I don’t feel I’ve changed the structure of my conversations … I’m not sure that there’s a real need. (HCP4)*

## Discussion

Whilst Covid-19 brought death and dying to the forefront - particularly for those identified as clinically extremely vulnerable, the results of this study indicate that the Covid-19 pandemic has had a limited impact on patient, BFCG and HCP experiences of engaging in EOLCP conversations. Patients and BFCG participants had varying levels of understanding around ESKD. Few patients or BFCG participants described experiences of engaging in EOLCP. The majority of patient and BFCG participants were unaware of the ReSPECT form and did not understand what this entailed as part of the EOLCP conversation. Many appeared to make sense of their illness in light of their faith. HCP participants described EOLCP as necessary but also as ‘difficult’, particularly when they were concerned about insufficient time to discuss EOLCP and resistance from patients and families. HCPs were reluctant to broach EOLCP and ReSPECT conversations unless the patient or their family did so. However, patients and families could mis-interpret a lack of conversation as implying the person’s health was stable and that such conversations were not needed. All HCP participants discussed the need for further training on a range of topics, including communication skills specifically related to EOLCP within the context of ESKD.

The results support previous studies which have suggested patients with kidney disease - including those across haemodialysis, peritoneal dialysis and conservative care pathways [14] and those from ethnically diverse backgrounds [15], often have a limited awareness and understanding of palliative and end-of-life care. The results of this study also support previous work which highlighted a lack of confidence amongst HCPs related to understanding the needs of and caring for patients from ED groups [16].

Patients with ESKD are reliant on their HCPs to ensure their end-of-life needs are anticipated and met [15]. Although HCPs report the importance of communicating issues related to end-of-life care, they also report varying levels of engagement in EOLCP. Here shared understanding and communication is key. The possible use of language translators to mediate discussions is helpful. However, even when consultations are conducted in English, it may be that patients and their family caregivers lack the fluency or cultural acclimatisation required to understand technical terms or pick up on the nuances of professional communication [16]. As such, only a very small minority of patients with ESKD report having the opportunity to engage in EOLCP [14].

The results presented suggest there is considerable scope to better support patients, family caregivers and HCPs to engage in EOLCP. For example, despite making sense of their illness in light of faith, participants tended not to discuss their spiritual or faith beliefs with HCPs. Framing discussions in light of someone’s spiritual or faith beliefs may provide an opportunity to discuss EOLCP. Furthermore, whilst the majority of patient and BFCG participants reported that their discussions with HCPs centred around the impact of lifestyle factors, EOLCP needs to be part of wider conversations which encompasses other aspects of living which puts the individual at risk of deterioration. If EOLCP discussions were re-framed to be included as part of broader supportive conversations around how to live well on dialysis, encompassing immediate and advance decisions at each stage of illness, it might lead to earlier engagement in EOLCP discussions. This shift - from a problem-orientated approach to a goal-orientated approach - may lead to better engagement, with more patient-centred outcomes [17].

There is a need to strengthen both cultural competence and cultural humility among individual HCPs and across health and social care organisations. Cultural humility reflects a recognition that understanding culture is not a fixed skill to be mastered but a lifelong, reflective process. It is typically defined as an ongoing commitment to critical self-reflection, acknowledging and addressing power imbalances, and recognising the limits of one’s own cultural perspective. Consequently, cultural humility involves more than simply eliciting a person’s understanding of their illness, prognosis, or future care options; it also requires situating these responses within the broader cultural, social, and structural contexts that shape people’s beliefs, experiences, and decisions [18]. It also requires situating these responses within broader cultural, social, and structural contexts that shape people’s beliefs, experiences, and decisions. One potential way to enhance cultural humility in practice is through HCPs adopting an “authentically curious approach” [16]. This involves the HCP and, where appropriate, the patient’s family or carers sharing their understanding of the illness, prognosis, and care options, and creating opportunities for all parties to express their concerns. The intention is to foster a shared space in which each can explore the other’s perspectives, expectations, and uncertainties. While valuable in all clinical encounters, such an approach becomes particularly important when HCPs are working with individuals or families whose cultural backgrounds differ from their own, and where assumptions or misunderstandings may be more likely to arise.

Whilst adopting this approach could be beneficial for all interactions, the need for such an approach may be heightened when working with patients and families with whom the HCP does not share a similar cultural background. An alternative way to enhance HCPs’ confidence and cultural humility may lie in the development of training for HCPs. However, enabling HCPs to undertake and utilise such training will also require a healthcare system which is sufficiently resourced. Further work is also needed to assess existing resources that are aimed at supporting EOLCP conversations (such as The Serious Illness Conversation Guide’ [19] and to consider the potential applicability of existing resources to be used or further tailored to support people from a range of cultural backgrounds and with a range of health conditions (including people living with ESKD).

It is widely recognised as best practice to involve patients, and/or their appointed proxy decision-makers, in the development of ReSPECT forms and similar documentation, particularly when the individual has reduced or fluctuating decision-making capacity [20]. Although meaningful involvement is central to the intent of the ReSPECT process, Hawkes et al., also found that documentation of patient or family involvement is not always consistently recorded, underscoring the need for more reliable engagement practices. Nevertheless, existing evidence indicates that some patients report being unaware of the existence of a ReSPECT form in their records. While this may often reflect limitations in patients’ recall or understanding, rather than the form being completed without their participation, it remains possible that such documentation can be established without the person’s knowledge [21]. Eli et al.,’s findings further demonstrate that when explanations are unclear or insufficient, patients and relatives may not fully comprehend the purpose or even the presence of the form, highlighting variability in practice and reinforcing the ongoing importance of ensuring transparent, patient‑centred conversations.

Research in the UK has suggested that families are often involved in decision-making with end stage kidney disease, yet families often feel uncertain about their role and unsupported to be involved in decision-making [22]. This highlights the need to consider how patients and their families — who frequently serve as informal sources of support and information — can be meaningfully empowered to make informed choices and decisions. There are on-going efforts to both measure patient involvement in decision-making in chronic kidney care [23] and to understand shared-decision making in the context of chronic kidney disease [24] and to develop evidence-based interventions to facilitate end-of-life decision-making involving patients with end stage kidney disease, their families and the healthcare professionals who support them [25]. Further research is needed to explore how families of people living with end stage kidney may be better supported to be involved in the completion of ReSPECT forms and other forms of advance care planning documentation.

Studies that have included non-English speakers also highlight the additional time and resources needed to organise translators, conduct the consultation and possibly increase the frequency of appointments to overcome communication barriers [26]. Central to this is a focus on both health literacy and death literacy, ensuring that patients and their family caregivers are equitably enabled to access, interpret, and apply information and services to support informed decision-making and action in relation to health, EOLC and EOLCP [26].

Despite heightened public awareness of mortality during the COVID-19 pandemic, its limited impact on end-of-life care planning in this study appears to reflect the persistence of pre-existing barriers, including competing clinical priorities, limited time, and healthcare professionals’ reluctance to initiate sensitive discussions unless prompted. For patients and families living with ESKD, immediate concerns related to treatment burden and day-to-day coping often took precedence over anticipatory planning. This suggests that increased awareness of death alone is insufficient to drive meaningful engagement in EOLCP without sustained changes in clinical practice.

Given the importance of faith in how many participants understood illness and prognosis, end-of-life care planning may be strengthened by routinely and explicitly inviting discussion of spiritual or religious beliefs using open, non-assumptive questions. Practical steps include greater integration of chaplaincy or community faith support and training for healthcare professionals that emphasises cultural humility. Embedding these approaches within routine dialysis care may help normalise conversations about future care and support more culturally responsive EOLCP.

The diversity of participants in the study is a strength (in terms of including a wide range of experiences and perspectives) but has some limitations in scope. The researchers attempted to include a broad range of ethnic diversity in the sample. However, not all groups are represented. In particular, the views of people from White minority groups – such as Jewish, Roma or Irish Traveller communities – are absent. The study was pragmatically limited in its geographical recruitment of patient and healthcare professional participants, who were all associated with one NHS Trust. However, we were able to recruit BFCGs from various regions across England, which enhanced the ethnic diversity of our sample and illuminated the shared experiences between patients and BFCGs.

## Conclusion

Despite Covid-19 bringing death and dying to the forefront of public discourse, ethnically diverse patients, bereaved family caregivers, and healthcare professionals report little change in their experiences with EOLCP. Patients and bereaved family carers continue to exhibit varying levels of understanding regarding prognosis in ESKD, likely due to limited discussions on future deterioration and EOLCP. While healthcare professionals recognise the importance of these conversations, they face significant challenges in practice. Therefore, targeted efforts are essential to enhance understanding and engagement in EOLCP discussions among dialysis patients from diverse ethnic backgrounds, fostering a more inclusive and effective approach.

## Supplementary Information

Below is the link to the electronic supplementary material.

Supplementary Material 1

## Abbreviations

BFCG: Bereaved family care giver
ED: Ethnically Diverse
EOLCP: End-of-life care planning
ESKD: End-stage kidney disease
HCP: Health Care Professional
PCBR: Public, carers and bereaved relatives
PPI: Patient and public involvement
ReSPECT: Recommended Summary Plan for Emergency Care and Treatment
