# Supplementary material for: End-of-life care planning for ethnically diverse kidney patients in the COVID-19 era
Source: BMC Nephrol. 2026 Mar 11;27:247. doi: 10.1186/s12882-026-04866-5 (PMC13094040; doi:10.1186/s12882-026-04866-5)
Supplement: Supplementary file 1 — Supplementary Material 1 [file 12882_2026_4866_MOESM1_ESM.docx]

**Interview Topic Guides**

**Prior to the interview complete consent and CRF**

- Date: Today is the (date) of (month), (year)
- Researchers name : My name is…
- Participants code: I am speaking to…
- Time interview starts: The time now is…

**Patient**

1. Experiences and understanding of ESKD (End Stage Kidney Disease)

- Could you tell me about your illness, how long have you had kidney disease?
- What is your understanding of end stage kidney disease and the future of your illness/prognosis?
- How has this impacted on your life, family etc?

2. Conversations with HCPs (Health care professionals)

Could you tell me about the sort of discussions you have with HCPs about your illness? (decision-making, treatment preferences, future care etc)

- Have you had/do you have conversations with any HCPs about future care if you were to become very ill, for example relating to the ReSPECT form? Is this form something you are aware of? If these conversations have taken place when have they happened? Who are these conversations with?
- Have you had conversations with HCPs about the risk COVID-19 might be to you as a kidney patient?
- Has COVID made any difference to the conversations you are having with HCPs about thinking ahead about your illness?

(explore positive/negative aspects of this).

3. Conversations with informal networks

- Are there people (groups, organisations, faith scholars, faith and community leaders) you draw upon for support or guidance? Are these people part of any formal support groups e.g. for kidney support etc.
- How important is your faith to you? How has your community supported you? Are there any particular faith leaders/schools of thought you follow?
- How did HCPs consider your faith when talking to you about the future?

**Health Care Professionals (HCPs)**

**Prior to the interview complete CRF**

- Date: Today is the (date) of (month), (year)
- Researchers name : My name is…
- Participants code: I am speaking to…
- Time interview starts: The time now is…

1. Experience of caring for patients with ESKD

Could you tell me about your interaction with patients with ESKD, how this happens here at Leicester General/Hamilton/Loughborough.

2. Conversations with patients and FCGs

Could you tell me about the sort of discussions you have with patients and FCG about ESKD (decision-making, treatment preferences, future care etc).

- Have you/do you have conversations with any patients/FCGs about future care if the patient were to become very ill, for example relating to the ReSPECT form? Is this form something you have completed? If these conversations have taken place when have they happened? Who are these conversations with – which HCPs and when?
- Have you had conversations about the risk COVID-19 specifically poses and how this might impact on ESKD patients?
- Do you think that since COVID conversations around end-of-life have changed/become more frequent/more regular/formalised?

(explore positive/negative aspects of this).

3. Conversations with informal networks

- Are there people (groups, organisations, faith scholars, faith and community leaders) you draw upon for support and guidance when having End of life care planning discussions with patients or FCGs?

**Bereaved Family Caregivers (BFCGs)**

**Prior to the interview complete CRF**

- Date: Today is the (date) of (month), (year)
- Researchers name : My name is…
- Participants code: I am speaking to…
- Time interview starts: The time now is…

1. Experience of supporting patient with ESKD.

Could you tell me about supporting your relative/friend with ESKD? How long had they been ill? What is your understanding of their illness and prognosis? How did this impact on your life, family etc?

2. Conversations with HCPs

Could you tell me about the sort of discussions you had with HCPs about your relatives/friends’ illness? (decision-making, treatment preferences, future care etc)

- Did you have conversations with any HCPs about future care if your relative/friend were to become very ill. For example relating to the ReSPECT form? Is this form something you were aware of? If these conversations took place when did they happen? Who were these conversations with?
- Did you have conversations about the risk COVID-19 might have posed to your friend/relative?
- Do you think COVID made a difference to the conversations you had with HCPs about thinking ahead about your friend/relative’s illness?

(explore positive/negative aspects of this).

3. Conversations with informal networks

- Are there people (groups, organisations, faith scholars, faith and community leaders) you draw upon for support or guidance? Are these people part of any formal support groups e.g. for kidney support, bereavement services etc.
- How important is your faith to you? How has your community supported you? Are there any particular faith leaders/schools of thought you follow?
- How did HCPs consider your friend/relative’s faith when thinking ahead about their illness?
